# Supplementary material for: Therapeutic Intervention with Anti-Complement Component 5 Antibody Does Not Reduce NASH but Does Attenuate Atherosclerosis and MIF Concentrations in Ldlr-/-.Leiden Mice
Source: Int J Mol Sci. 2022 Sep 14;23(18):10736. doi: 10.3390/ijms231810736 (PMC9506266; doi:10.3390/ijms231810736)
Supplement: Supplementary file 1 [file ijms-23-10736-s001.zip › ijms-1864006-supplementary.pdf]

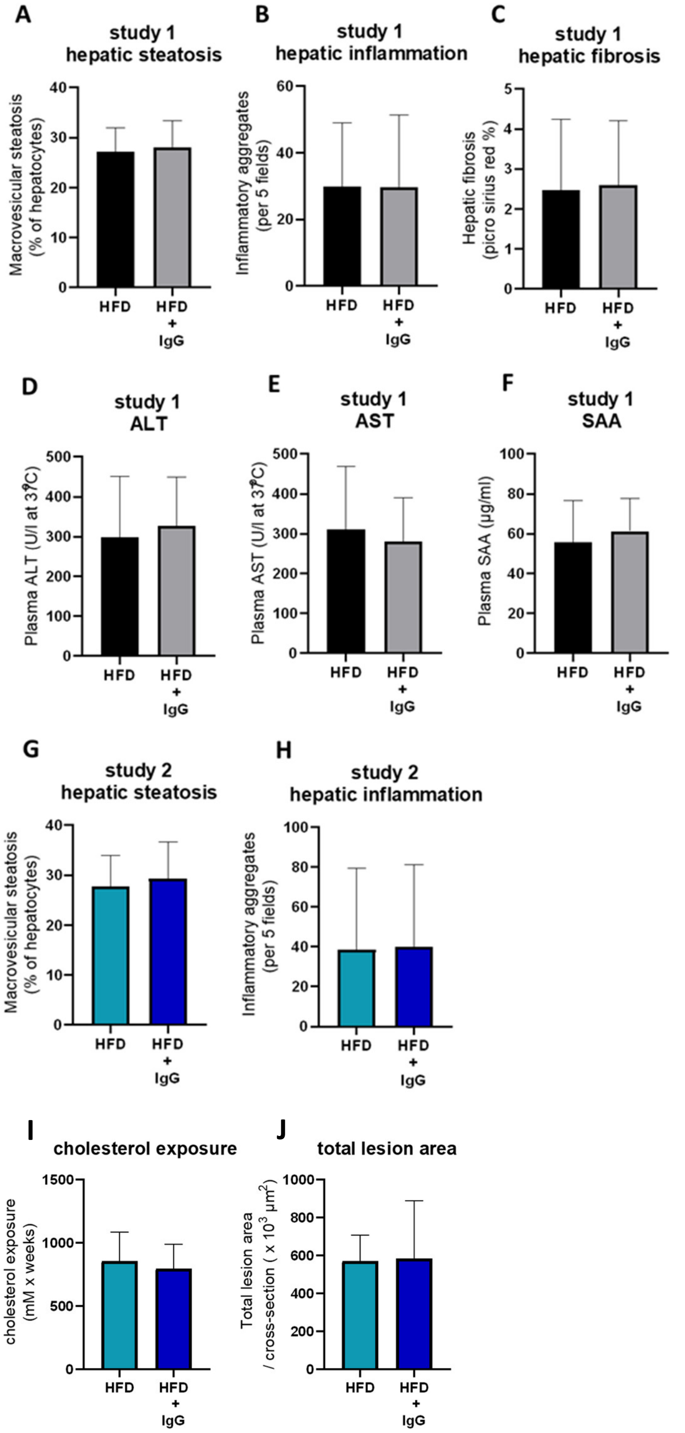

Supplemental Figure S1: IgG isotype control treatment does not affect the development of NASH in high-fat diet (HFD)-fed Ldlr-/-.Leiden mice. Data is shown from two independent studies. Study 1 (panels A-F): Ldlr-/-.Leiden mice (n=15 per group) were fed HFD for 38 weeks, of which the last 16 weeks included IgG isotype control treatment. IgG isotype control treatment did not affect histologically assessed (by board-certified pathologist) hepatic steatosis (A), hepatic

inflammation (B) or hepatic fibrosis (C) relative to untreated HFD controls. In addition, IgG treatment did not affect plasma levels of the liver enzymes alanine aminotransferase (ALT, panel D) and aspartate aminotransferase (AST, panel E), or the systemic inflammation marker serum amyloid A (SAA, panel F). Study 2 (panels G-H): Ldlr /-.Leiden mice (n=15 per group) were fed HFD for 33 weeks, of which the last 13 weeks included IgG isotype control treatment. Histopathological analysis of NASH (by board-certified pathologist) confirmed that hepatic steatosis (G) and hepatic inflammation (H) were not affected by treatment with an IgG isotype control. N.B. study 2 focused on hepatic inflammation as the primary endpoint. Hepatic fibrosis and plasma liver enzymes / inflammation markers were not analyzed in this study. The IgG isotype control-treated animals from this study also had a cholesterol exposure (I) and atherosclerosis development (histological analysis aortic root) (J) comparable to untreated HFD controls. Data shown represent mean ± standard deviation.

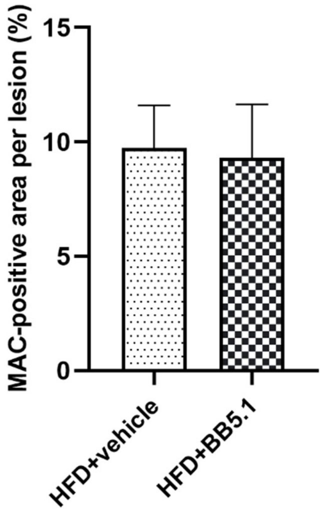

Supplemental Figure S2: Anti-C5 treatment did not reduce the proportion of MAC-positive area per atherosclerotic lesion. Data are mean ± SEM. * $p \leq 0.05$, ** $p \leq 0.01$, *** $p \leq 0.001$ HFD+vehicle vs. HFD+BB5.1

Supplemental table S1: Canonical pathways analysis based on hepatic gene expression in HFD+vehicle vs Chow. The Z-score indicates the predicted activation of a canonical pathway: Z-score ≤ -2 indicates relevant inhibition of the pathway (shown in green); Z-score ≥ 2 indicates relevant activation of the pathway (shown in orange). P-value ≤ 0.01 (-log(P-value) ≥ 2).indicates significant enrichment of the genes involved in the canonical pathway.

Canonical pathway	Activation Z-score	- log (P-value)
Phagosome Formation	N/A	14,06
Hepatic Fibrosis Signaling Pathway	5,77	13,90
FcÎ³ Receptor-mediated Phagocytosis in Macrophages and Monocytes	5,55	13,69
PI3K Signaling in B Lymphocytes	6,02	13,67
Neuroinflammation Signaling Pathway	5,88	13,36
Integrin Signaling	5,74	12,97
GP6 Signaling Pathway	5,35	12,64
IL-8 Signaling	6,35	11,96
CD28 Signaling in T Helper Cells	5,01	11,64
Role of NFAT in Regulation of the Immune Response	5,91	11,31
Cardiac Hypertrophy Signaling (Enhanced)	6,12	10,75
Axonal Guidance Signaling	N/A	10,68
Hepatic Fibrosis / Hepatic Stellate Cell Activation	N/A	10,62
iCOS-iCOSL Signaling in T Helper Cells	4,84	10,61
Tec Kinase Signaling	5,63	10,54
Caveolar-mediated Endocytosis Signaling	N/A	10,52
Macropinocytosis Signaling	3,78	10,48
Dendritic Cell Maturation	6,45	9,97
GÎ±q Signaling	3,87	9,95
Systemic Lupus Erythematosus In B Cell Signaling Pathway	6,53	9,78
HER-2 Signaling in Breast Cancer	4,36	9,59
Rac Signaling	5,73	9,41
Signaling by Rho Family GTPases	6,84	9,40
Actin Nucleation by ARP-WASP Complex	4,43	9,34
Production of Nitric Oxide and Reactive Oxygen Species in Macrophages	5,01	9,34
Role of Macrophages, Fibroblasts and Endothelial Cells in Rheumatoid Arthritis	N/A	9,26
Phospholipase C Signaling	5,34	9,20
fMLP Signaling in Neutrophils	5,29	9,18
Glioma Invasiveness Signaling	3,77	9,16
Leukocyte Extravasation Signaling	4,59	8,86
B Cell Receptor Signaling	5,66	8,85
NF-Î°B Signaling	5,42	8,85
T Cell Receptor Signaling	N/A	8,71

Senescence Pathway	3,92	8,69
FAK Signaling	N/A	8,50
Ephrin Receptor Signaling	4,53	8,50
Sirtuin Signaling Pathway	-0,22	8,27
Molecular Mechanisms of Cancer	N/A	8,26
Glioma Signaling	4,22	8,06
Sphingosine-1-phosphate Signaling	3,32	7,99
Superpathway of Cholesterol Biosynthesis	-3,44	7,94
LXR/RXR Activation	-3,90	7,90
p70S6K Signaling	4,04	7,72
Apoptosis Signaling	0,47	7,57
Actin Cytoskeleton Signaling	4,56	7,57
HGF Signaling	4,11	7,56
HIF1Î± Signaling	5,12	7,55
UVA-Induced MAPK Signaling	3,78	7,38
Neuregulin Signaling	2,83	7,31
Virus Entry via Endocytic Pathways	N/A	7,26
FXR/RXR Activation	N/A	7,20
Regulation of Cellular Mechanics by Calpain Protease	1,28	7,11
ERK/MAPK Signaling	4,11	7,11
Acute Phase Response Signaling	3,05	7,10
P2Y Purigenic Receptor Signaling Pathway	3,75	7,07
LPS/IL-1 Mediated Inhibition of RXR Function	1,92	7,05
Xenobiotic Metabolism Signaling	N/A	6,99
Th1 Pathway	3,80	6,96
TREM1 Signaling	4,23	6,93
Estrogen-Dependent Breast Cancer Signaling	3,53	6,93
Th1 and Th2 Activation Pathway	N/A	6,90
Ceramide Signaling	1,15	6,89
T Cell Exhaustion Signaling Pathway	1,61	6,86
Apelin Endothelial Signaling Pathway	4,42	6,84
PTEN Signaling	-3,09	6,82
Tumor Microenvironment Pathway	4,25	6,76
NF-Î°B Activation by Viruses	4,35	6,76
Role of Tissue Factor in Cancer	N/A	6,71
GM-CSF Signaling	4,13	6,70
mTOR Signaling	4,43	6,70
PI3K/AKT Signaling	3,04	6,69
Role of NFAT in Cardiac Hypertrophy	4,28	6,68
PKCÎ¸ Signaling in T Lymphocytes	5,91	6,67
14-3-3-mediated Signaling	5,03	6,63
Colorectal Cancer Metastasis Signaling	5,50	6,60
Thrombin Signaling	4,74	6,54
Mitochondrial Dysfunction	N/A	6,52

Role of Pattern Recognition Receptors in Recognition of Bacteria and Viruses	4,96	6,39
Endothelin-1 Signaling	3,75	6,30
Toll-like Receptor Signaling	2,56	6,21
Altered T Cell and B Cell Signaling in Rheumatoid Arthritis	N/A	6,08
Renin-Angiotensin Signaling	3,68	6,01
Neuropathic Pain Signaling In Dorsal Horn Neurons	3,28	5,97
Coagulation System	0,00	5,96
Paxillin Signaling	4,32	5,95
IL-6 Signaling	4,62	5,90
RhoGDI Signaling	-5,22	5,86
Protein Kinase A Signaling	1,57	5,85
Opioid Signaling Pathway	2,43	5,84
Natural Killer Cell Signaling	4,52	5,82
Adrenomedullin signaling pathway	4,50	5,82
CXCR4 Signaling	4,81	5,81
MSP-RON Signaling In Cancer Cells Pathway	3,39	5,81
CTLA4 Signaling in Cytotoxic T Lymphocytes	N/A	5,74
Regulation of Actin-based Motility by Rho	4,75	5,70
Thrombopoietin Signaling	3,40	5,70
STAT3 Pathway	3,00	5,70
Th2 Pathway	2,47	5,59
Chemokine Signaling	4,24	5,59
Amyotrophic Lateral Sclerosis Signaling	2,40	5,58
Acetone Degradation I (to Methylglyoxal)	-2,83	5,56
Ferroptosis Signaling Pathway	1,90	5,50
Estrogen Receptor Signaling	2,42	5,49
Cardiac Hypertrophy Signaling	5,18	5,47
Growth Hormone Signaling	2,19	5,45
Death Receptor Signaling	3,33	5,34
PEDF Signaling	2,56	5,30
Glioblastoma Multiforme Signaling	4,71	5,29
Acute Myeloid Leukemia Signaling	1,98	5,28
Granulocyte Adhesion and Diapedesis	N/A	5,25
IL-3 Signaling	3,89	5,25
PDGF Signaling	4,80	5,22
Role of Hypercytokinemia/hyperchemokinemia in the Pathogenesis of Influenza	4,90	5,22
Osteoarthritis Pathway	2,29	5,20
Regulation of eIF4 and p70S6K Signaling	3,14	5,20
T Helper Cell Differentiation	N/A	5,14
Reelin Signaling in Neurons	5,03	5,14
Systemic Lupus Erythematosus Signaling	N/A	5,11

Role of Osteoblasts, Osteoclasts and Chondrocytes in Rheumatoid Arthritis	N/A	5,05
G-Protein Coupled Receptor Signaling	N/A	5,04
Estrogen Biosynthesis	-2,84	4,99
Oxidative Phosphorylation	-6,40	4,98
Superpathway of Citrulline Metabolism	-1,51	4,98
Sperm Motility	3,00	4,97
HMGB1 Signaling	5,60	4,95
PAK Signaling	4,38	4,92
PD-1, PD-L1 cancer immunotherapy pathway	-1,62	4,92
Semaphorin Neuronal Repulsive Signaling Pathway	-0,14	4,91
Nitric Oxide Signaling in the Cardiovascular System	2,67	4,90
Xenobiotic Metabolism General Signaling Pathway	2,26	4,89
NRF2-mediated Oxidative Stress Response	2,35	4,87
Cholesterol Biosynthesis II (via 24,25-dihydrolanosterol)	-3,16	4,86
Cholesterol Biosynthesis I	-3,16	4,86
Cholesterol Biosynthesis III (via Desmosterol)	-3,16	4,86
Angiopoietin Signaling	1,96	4,86
Communication between Innate and Adaptive Immune Cells	N/A	4,84
Cell Cycle: G2/M DNA Damage Checkpoint Regulation	0,23	4,82
Hereditary Breast Cancer Signaling	N/A	4,81
Citrulline Biosynthesis	-0,71	4,78
Regulation of IL-2 Expression in Activated and Anergic T Lymphocytes	N/A	4,71
LPS-stimulated MAPK Signaling	4,13	4,71
JAK/Stat Signaling	2,69	4,65
Clathrin-mediated Endocytosis Signaling	N/A	4,56
Aryl Hydrocarbon Receptor Signaling	2,60	4,54
Prolactin Signaling	2,92	4,52
Apelin Cardiomyocyte Signaling Pathway	3,78	4,49
ILK Signaling	4,18	4,49
IL-4 Signaling	N/A	4,46
IL-2 Signaling	4,08	4,45
Induction of Apoptosis by HIV1	1,80	4,45
Chronic Myeloid Leukemia Signaling	N/A	4,45
Germ Cell-Sertoli Cell Junction Signaling	N/A	4,44
IL-12 Signaling and Production in Macrophages	N/A	4,41
Mevalonate Pathway I	-1,67	4,40
Cdc42 Signaling	4,35	4,36
Stearate Biosynthesis I (Animals)	-1,41	4,27
Coronavirus Pathogenesis Pathway	3,00	4,26
ErbB Signaling	4,35	4,24

Urea Cycle	-1,63	4,24
PPAR Signaling	-3,78	4,24
Renal Cell Carcinoma Signaling	4,26	4,21
Pancreatic Adenocarcinoma Signaling	3,41	4,20
Bupropion Degradation	-2,67	4,20
Ubiquinol-10 Biosynthesis (Eukaryotic)	-1,51	4,19
Relaxin Signaling	3,18	4,18
MSP-RON Signaling In Macrophages Pathway	0,00	4,18
Glycine Betaine Degradation	-2,83	4,16
Lymphotoxin Î² Receptor Signaling	3,58	4,15
Superpathway of Inositol Phosphate Compounds	3,20	4,12
ErbB4 Signaling	3,55	4,08
HOTAIR Regulatory Pathway	2,94	4,08
Inflammasome pathway	2,89	4,07
Histidine Degradation VI	-1,27	4,01
Endocannabinoid Developing Neuron Pathway	2,33	3,98
Cholecystokinin/Gastrin-mediated Signaling	4,44	3,96
TWEAK Signaling	1,21	3,94
Gap Junction Signaling	N/A	3,91
Hepatic Cholestasis	N/A	3,91
Thyroid Cancer Signaling	2,92	3,90
PPARÎ±/RXRÎ± Activation	-1,94	3,84
Insulin Secretion Signaling Pathway	2,26	3,84
Role of JAK1 and JAK3 in Î³c Cytokine Signaling	N/A	3,83
EIF2 Signaling	-1,04	3,80
Glucocorticoid Receptor Signaling	N/A	3,80
FLT3 Signaling in Hematopoietic Progenitor Cells	3,78	3,79
Inhibition of Matrix Metalloproteases	-1,00	3,78
IL-15 Production	3,28	3,78
Calcium-induced T Lymphocyte Apoptosis	3,00	3,76
B Cell Development	N/A	3,75
VEGF Signaling	3,18	3,72
IL-10 Signaling	N/A	3,70
Telomerase Signaling	3,02	3,67
Type I Diabetes Mellitus Signaling	3,40	3,65
3-phosphoinositide Biosynthesis	2,38	3,62
IL-7 Signaling Pathway	3,92	3,61
Crosstalk between Dendritic Cells and Natural Killer Cells	4,71	3,61
RANK Signaling in Osteoclasts	3,78	3,61
TNFR1 Signaling	2,68	3,60
Melanoma Signaling	3,44	3,60
Primary Immunodeficiency Signaling	N/A	3,60
Small Cell Lung Cancer Signaling	2,68	3,59

CNTF Signaling	3,41	3,58
Ovarian Cancer Signaling	2,27	3,57
Apelin Adipocyte Signaling Pathway	-0,76	3,57
IL-15 Signaling	3,78	3,54
FcÎ³RIIB Signaling in B Lymphocytes	3,55	3,54
Î±-tocopherol Degradation	0,00	3,53
Folate Polyglutamylation	-1,34	3,53
Lysine Degradation II	-2,24	3,53
Lysine Degradation V	-2,24	3,53
Melatonin Signaling	1,18	3,47
AMPK Signaling	1,07	3,47
Fc Epsilon RI Signaling	4,00	3,46
SAPK/JNK Signaling	3,31	3,44
GNRH Signaling	3,73	3,42
D-myo-inositol-5-phosphate Metabolism	2,31	3,41
Prostate Cancer Signaling	N/A	3,41
Î±-Adrenergic Signaling	2,84	3,38
EGF Signaling	2,84	3,38
RAR Activation	N/A	3,38
Sertoli Cell-Sertoli Cell Junction Signaling	N/A	3,38
VEGF Family Ligand-Receptor Interactions	3,53	3,36
Non-Small Cell Lung Cancer Signaling	3,27	3,36
iNOS Signaling	2,84	3,35
Type II Diabetes Mellitus Signaling	4,33	3,34
Glutathione-mediated Detoxification	-1,81	3,33
Tryptophan Degradation III (Eukaryotic)	-1,51	3,29
Atherosclerosis Signaling	N/A	3,28
GÎ±12/13 Signaling	3,04	3,28
CREB Signaling in Neurons	6,59	3,26
Leptin Signaling in Obesity	3,05	3,25
CDK5 Signaling	2,48	3,24
FAT10 Cancer Signaling Pathway	2,83	3,20
Huntington's Disease Signaling	2,78	3,18
IL-9 Signaling	2,32	3,16
Necroptosis Signaling Pathway	3,75	3,13
TNFR2 Signaling	1,94	3,12
p53 Signaling	-1,40	3,11
Ketolysis	0,82	3,11
Superpathway of Geranylgeranyldiphosphate Biosynthesis I (via Mevalonate)	-1,67	3,10
ERK5 Signaling	3,27	3,07
Role of PKR in Interferon Induction and Antiviral Response	4,00	3,06
CD40 Signaling	2,56	3,04

Xenobiotic Metabolism PXR Signaling Pathway	-3,21	3,00
Inhibition of Angiogenesis by TSP1	2,53	2,99
Wnt/Ca+ pathway	1,88	2,97
Pregnenolone Biosynthesis	-0,38	2,95
Bile Acid Biosynthesis, Neutral Pathway	-2,83	2,95
Synaptogenesis Signaling Pathway	6,04	2,95
Superoxide Radicals Degradation	0,00	2,95
Histidine Degradation III	-1,63	2,95
Antiproliferative Role of Somatostatin Receptor 2	3,27	2,95
Agranulocyte Adhesion and Diapedesis	N/A	2,94
IGF-1 Signaling	2,35	2,93
Xenobiotic Metabolism CAR Signaling Pathway	-2,02	2,93
CCR3 Signaling in Eosinophils	4,20	2,91
Docosahexaenoic Acid (DHA) Signaling	N/A	2,89
UVB-Induced MAPK Signaling	3,30	2,88
Iron homeostasis signaling pathway	N/A	2,84
Zymosterol Biosynthesis	-2,24	2,83
Acetate Conversion to Acetyl-CoA	-1,00	2,82
Endometrial Cancer Signaling	3,13	2,79
Melatonin Degradation I	-3,41	2,79
BEX2 Signaling Pathway	1,73	2,76
Ketogenesis	0,00	2,75
Regulation Of The Epithelial Mesenchymal Transition By Growth Factors Pathway	5,55	2,75
Antigen Presentation Pathway	N/A	2,74
Nicotine Degradation III	-3,27	2,72
Amyloid Processing	1,94	2,69
GDNF Family Ligand-Receptor Interactions	3,13	2,68
Ethanol Degradation IV	-0,30	2,66
TR/RXR Activation	N/A	2,65
Phospholipases	1,96	2,65
Nicotine Degradation II	-3,13	2,65
PXR/RXR Activation	N/A	2,65
Superpathway of Melatonin Degradation	-3,13	2,65
Erythropoietin Signaling Pathway	2,26	2,65
nNOS Signaling in Neurons	2,45	2,62
April Mediated Signaling	2,32	2,61
Breast Cancer Regulation by Stathmin1	4,93	2,60
Synaptic Long Term Potentiation	3,33	2,56
Pyridoxal 5'-phosphate Salvage Pathway	3,41	2,55
Folate Transformations I	0,00	2,55
Kinetochore Metaphase Signaling Pathway	3,29	2,55
Triacylglycerol Biosynthesis	-0,26	2,55
IL-23 Signaling Pathway	3,00	2,55

Antioxidant Action of Vitamin C	-3,16	2,55
Agrin Interactions at Neuromuscular Junction	3,44	2,53
G Beta Gamma Signaling	3,33	2,49
B Cell Activating Factor Signaling	2,89	2,48
Tumoricidal Function of Hepatic Natural Killer Cells	1,89	2,48
Glutathione Redox Reactions I	-0,91	2,48
NGF Signaling	3,66	2,48
CCR5 Signaling in Macrophages	3,00	2,48
Phagosome Maturation	N/A	2,48
Endocannabinoid Cancer Inhibition Pathway	-0,62	2,46
Glucose and Glucose-1-phosphate Degradation	1,89	2,45
3-phosphoinositide Degradation	1,81	2,43
RhoA Signaling	4,23	2,42
Role of PI3K/AKT Signaling in the Pathogenesis of Influenza	2,68	2,38
Remodeling of Epithelial Adherens Junctions	3,46	2,37
Nur77 Signaling in T Lymphocytes	2,29	2,37
Trehalose Degradation II (Trehalase)	2,24	2,36
Glutamate Receptor Signaling	-0,38	2,33
IL-1 Signaling	1,21	2,33
D-myo-inositol (1,4,5)-Trisphosphate Biosynthesis	2,11	2,31
ErbB2-ErbB3 Signaling	3,58	2,29
Myc Mediated Apoptosis Signaling	1,41	2,28
Triacylglycerol Degradation	-1,41	2,28
Xenobiotic Metabolism AHR Signaling Pathway	-0,58	2,26
Adipogenesis pathway	N/A	2,25
Oncostatin M Signaling	3,36	2,24
MSP-RON Signaling Pathway	N/A	2,23
Cancer Drug Resistance By Drug Efflux	N/A	2,23
Methylglyoxal Degradation III	-1,00	2,23
Oxidative Ethanol Degradation III	-0,33	2,23
Interferon Signaling	2,67	2,22
p38 MAPK Signaling	3,02	2,22
Ephrin A Signaling	N/A	2,21
Glutaryl-CoA Degradation	-0,38	2,20
Chondroitin Sulfate Degradation (Metazoa)	1,41	2,20
Tyrosine Degradation I	-2,00	2,20
UDP-N-acetyl-D-galactosamine Biosynthesis II	1,89	2,20
Melanocyte Development and Pigmentation Signaling	2,50	2,18
Epithelial Adherens Junction Signaling	N/A	2,18
Glycolysis I	2,71	2,16
Activation of IRF by Cytosolic Pattern Recognition Receptors	1,09	2,13
Thymine Degradation	N/A	2,12

Methionine Salvage II (Mammalian)	N/A	2,12
Uracil Degradation II (Reductive)	N/A	2,12
NADH Repair	N/A	2,12
Regulation of the Epithelial-Mesenchymal Transition Pathway	N/A	2,11
Superpathway of Methionine Degradation	-3,21	2,10
Dopamine-DARPP32 Feedback in cAMP Signaling	2,53	2,05
Semaphorin Signaling in Neurons	N/A	2,05
Valine Degradation I	-2,12	2,05
D-myo-inositol (3,4,5,6)-tetrakisphosphate Biosynthesis	1,44	2,04
D-myo-inositol (1,4,5,6)-Tetrakisphosphate Biosynthesis	1,44	2,04
Ephrin B Signaling	2,18	2,03
Role of BRCA1 in DNA Damage Response	2,00	2,03
Neurotrophin/TRK Signaling	2,99	2,03
Cytotoxic T Lymphocyte-mediated Apoptosis of Target Cells	2,71	2,02
Isoleucine Degradation I	-0,82	2,00

Supplemental Table S2: Differential regulation of genes in the 'oxidative phosphorylation' pathway. Gene expression changes are expressed as 2log fold-change (2logR), -log(P-value) ≥ 2 indicates significant differential expression of a gene, green color indicating significant downregulation and orange color indicating significant upregulation.

	HFD+vehicle vs. Chow		*HFD+BB5.1 vs. HFD+vehicle*	
**Genes**	**Fold change (2logR)**	**- log (P-value)**	**Fold change (2logR)**	**- log (P-value)**
MT-ND1	-0,46	8,30	0,18	2,30
NDUFB11	-0,51	6,91	0,17	1,49
NDUFS7	-0,60	6,65	0,25	1,97
ATP5F1D	-0,41	6,60	0,10	0,90
ATP5F1C	-0,27	6,31	0,05	0,63
NDUFB5	-0,39	5,21	0,09	0,69
NDUFS6	-1,70	5,11	0,08	0,08
CYB5A	-0,42	5,10	0,15	1,25
SDHB	-0,41	4,70	0,12	0,91
ATP5PD	-0,42	4,54	0,12	0,81
CYC1	-0,28	4,47	0,05	0,40
NDUFS3	-0,34	4,36	0,13	1,20
NDUFAB1	-0,36	3,90	0,22	2,34
UQCRFS1	-0,30	3,86	0,10	0,85
MT-CO1	-0,35	3,74	0,16	1,41
UQCR11	-0,49	3,61	0,20	1,14
NDUFA6	-0,38	3,47	0,12	0,73
NDUFB10	-0,35	3,29	0,07	0,37
SURF1	-0,33	3,24	0,07	0,45
SDHC	-0,19	3,22	0,07	0,83
NDUFA9	-0,24	3,12	0,08	0,79
NDUFA12	-0,37	3,05	0,27	2,48
NDUFV2	-0,26	2,96	0,13	1,32
NDUFB6	-0,34	2,86	0,12	0,74
ATP5PO	-0,73	2,78	0,13	0,30
NDUFB4	-0,35	2,66	0,23	1,82
MT-ND2	-0,25	2,53	0,16	1,68
NDUFB3	-0,31	2,40	0,20	1,54
ATP5MF	-0,36	2,37	0,21	1,33
NDUFB7	-0,36	2,31	0,28	2,09
NDUFA11	-0,35	2,31	0,20	1,30
UQCRC1	-0,24	2,30	0,07	0,48
NDUFS8	-0,27	2,28	0,14	1,10
ATP5PF	-0,32	2,20	0,14	0,79
ATP5PB	-0,14	2,09	0,09	1,52
NDUFV3	-0,29	2,08	0,02	0,08

COX5A	-0,20	2,06	0,19	2,42
SDHD	-0,19	2,05	0,06	0,51
NDUFA7	-0,32	2,04	0,23	1,69
COX7B	-0,28	2,02	0,21	1,70
NDUFB2	-0,52	2,01	-0,14	0,37
UQCRB	-0,30	2,00	0,27	2,37
ATP5MG	-0,31	1,78	0,30	2,26
UQCRQ	-0,27	1,34	0,32	2,38
NDUFA4	-0,20	1,22	0,24	2,13
MT-CYB	0,24	1,17	0,15	0,77
COX6A1	-0,22	1,09	0,29	2,13
NDUFA5	-0,25	1,05	0,36	2,49
